# Relationship Between the Duration of Intravenous Ketamine Anesthesia and Postoperative Oxidative Stress and Inflammatory Response in Rats

**DOI:** 10.3390/ijms26199465

**Published:** 2025-09-27

**Authors:** Ramazan Ince, Habip Burak Ozgodek, Agah Abdullah Kahramanlar, Nurinisa Yucel, Cengiz Sarıgül, Halis Suleyman

**Affiliations:** 1 Department of Anesthesiology and Reanimation, Erzurum City Hospital, 25240 Erzurum, Turkey; 2 Pharmacy Services Program, Vocational School of Health Services, Erzincan Binali Yildirim University, 24100 Erzincan, Turkey; 3 Department of Medical Biochemistry, Faculty of Medicine, Erzincan Binali Yildirim University, 24100 Erzincan, Turkey; 4 Department of Pharmacology, Faculty of Medicine, Erzincan Binali Yildirim University, 24100 Erzincan, Turkey

**Keywords:** anesthesia duration, inflammation, ketamine, laparotomy, oxidative stress

## Abstract

Surgical trauma triggers oxidative and inflammatory responses that contribute to postoperative complications. Although the antioxidant and anti-inflammatory effects of ketamine have been reported, the impact of anesthesia duration on these mechanisms remains unclear. Forty-two male Wistar rats were randomized into healthy control (HG), ketamine only (KET; 60 mg/kg, i.p.), or laparotomy plus ketamine with 0–4 additional ketamine doses at 20 min intervals (KET + L, KET + L1–L4). At 24 h, levels of MDA, tGSH, SOD, CAT, IL-1β, IL-6, TNF-α, adrenaline and noradrenaline were measured in tail-vein blood. One-way ANOVA with Tukey’s post hoc test was used. Laparotomy under single-dose ketamine increased MDA and pro-inflammatory cytokines and decreased tGSH, SOD, CAT, ADR, and NDR versus HG and KET (all *p* < 0.001). After laparotomy, repeated ketamine dosing produced graded decreases in MDA and cytokines and increases in tGSH, SOD, CAT, ADR, and NDR toward control levels; effects were most pronounced in KET + L4 (all *p* < 0.001). Ketamine alone did not differ significantly from HG. In rats, ketamine modulates postoperative biological stress in a duration-dependent manner; prolonging anesthesia reduces oxidative–inflammatory load and restores catecholaminergic tone. These findings strongly support revisiting dose–duration protocols and underscore the need for mechanistic and clinical studies.

## 1. Introduction

Surgical trauma elicits a stress response that leads to marked changes in the neuroendocrine, inflammatory, hemodynamic, and metabolic systems following surgical interventions [1]. This response may be accompanied by serious, life-threatening complications [2]. Although many factors contribute to these complications, one of the most clinically notable mechanisms is the induction of oxidative stress [1,3]. Oxidative stress is driven by excessive production of reactive oxygen species (ROS). Uncontrolled increases in ROS suppress cellular antioxidant defense systems and cause destructive events such as lipid peroxidation (LPO) in cell membranes, protein denaturation, and DNA damage [4]. These events both increase the risk of postoperative complications and adversely affect tissue healing processes [5]. Indeed, various clinical studies have reported important findings indicating that reducing oxidative stress accelerates postoperative recovery and lowers complication rates [6,7]. In a previous study, significant increases in ROS levels were detected in the postoperative period in patients undergoing coronary artery bypass grafting, and this increase was associated with the development of postoperative myocardial infarction [8]. Similarly, Wu and colleagues reported that patients with higher postoperative oxidative-stress biomarker levels had a three-fold increased risk of atrial fibrillation [9].

Hemodynamic instability after surgical intervention is among the important causes of morbidity and mortality in patients monitored in intensive care units [10]. In this context, postoperative hypotension is frequently encountered yet often unrecognized [11]. This condition can lead to multisystem dysfunctions such as myocardial infarction, acute kidney injury, cerebrovascular events, and delirium [11]. Retrospective analyses have shown that a mean arterial pressure (MAP) below 65 mmHg is associated with these complications [12]. Studies have suggested that oxidative stress and the inflammatory response also play a central role in the pathogenesis of multi-organ injury caused by postoperative hypotension [1]. Therefore, anesthetic agents used in the perioperative period are critically important not only for intraoperative stability but also for the prevention of postoperative complications.

Moreover, the anesthesia techniques employed can significantly affect oxidative-stress levels in the perioperative period [13]. Anesthesia enables surgical procedures to be performed safely, rapidly, and effectively by providing fundamental physiological conditions such as analgesia, loss of consciousness, and adequate muscle relaxation [14]. However, anesthetic agents are not limited to these pharmacological effects; they may also shape the oxidative-stress and inflammatory responses that develop due to surgical trauma [15]. There is an increasing body of evidence that intravenously administered anesthetic agents can modulate the oxidant–antioxidant balance at the cellular level [16].

In our study, which aimed to investigate the relationship between anesthesia duration and surgical-trauma-induced oxidative stress, inflammatory response, and catecholamine levels, the agent used was ketamine, a phencyclidine-derived intravenous anesthetic [17]. Widely used in clinical practice, ketamine exerts its central nervous system effects through N-methyl-D-aspartate (NMDA) receptor antagonism [18]. Beyond its analgesic effects, various clinical and preclinical studies have suggested that it may exhibit anti-inflammatory and antioxidant properties [19]. As shown by Liang and colleagues reported that ketamine significantly reduced the increase in malondialdehyde (MDA) levels in brain tissue and prevented apoptosis by increasing superoxide dismutase (SOD) activity [20]. In addition, the ability of ketamine to increase endogenous catecholamine production [21] is indicative of its sympathomimetic activity. Furthermore, some studies have shown that ketamine suppresses increases in interleukin-1 beta (IL-1β), interleukin-6 (IL-6), and tumor necrosis factor-alpha (TNF-α) levels and attenuates hypotension [22,23]. These findings suggest that ketamine may be a potential therapeutic agent for maintaining hemodynamic, oxidative, and inflammatory balance. However, how these effects vary with anesthesia duration remains unclear. To date, no comprehensive study has directly compared the effects of short- and long-term ketamine use on surgical-trauma-related oxidative stress and inflammatory responses. Therefore, the aim of this study is to investigate the relationship between the duration of intravenous ketamine anesthesia and postoperative-trauma-related oxidative stress, inflammatory response, and catecholamine levels in rats.

## 2. Results

### 2.1. Blood MDA and tGSH (Total Glutathione) Analysis Results

**Figure 1:**
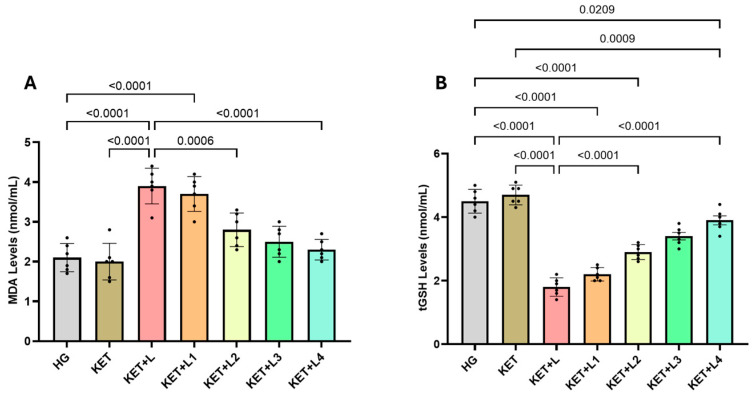
Blood levels of MDA (**A**) and tGSH (**B**) in the experimental groups. Bars indicate mean ± standard deviation (SD); *n* = 6 per group. Black dots represent individual animal values. Statistical comparisons between groups were performed using one-way ANOVA followed by Tukey’s post hoc test. Exact *p*-values are shown above the bars. Statistical significance was set at *p* < 0.05. Abbreviations: MDA, malondialdehyde; tGSH, total glutathione; HG, healthy group; KET, ketamine group; KET + L, laparotomy + ketamine group; KET + L1–L4, laparotomy + repeated ketamine dose groups.

In tail-vein blood samples, MDA levels (nmol/mL) were significantly increased in the group that underwent laparotomy in addition to anesthesia (KET + L; 3.90 ± 0.45) compared with the healthy control (HG; 2.10 ± 0.36) and the anesthesia-only group (KET; 2.00 ± 0.46) (*p* < 0.0001 vs. both). While MDA levels remained elevated in the group receiving one additional dose (KET + L1; 3.70 ± 0.44) (*p* < 0.05), significant decreases were observed compared with KET + L in the groups receiving two (KET + L2; 2.80 ± 0.42), three (KET + L3; 2.50 ± 0.39), and four (KET + L4; 2.30 ± 0.26) additional ketamine doses. Notably, values in the KET + L4 group approached control levels (all *p* < 0.001) (Figure 1A, Table 1).

tGSH levels (nmol/mL) in tail-vein blood were markedly lower in KET + L (1.80 ± 0.29) compared with HG (4.50 ± 0.37) and KET (4.70 ± 0.31) (*p* < 0.0001). While tGSH in KET + L1 (2.20 ± 0.21) did not differ significantly from KET + L (*p* > 0.05), a stepwise increase was detected compared with KET + L in KET + L2 (2.90 ± 0.24), KET + L3 (3.40 ± 0.29), and KET + L4 (3.90 ± 0.35) (all *p* < 0.001) (Figure 1B, Table 1).

### 2.2. Blood SOD and CAT Activity Results

**Figure 2:**
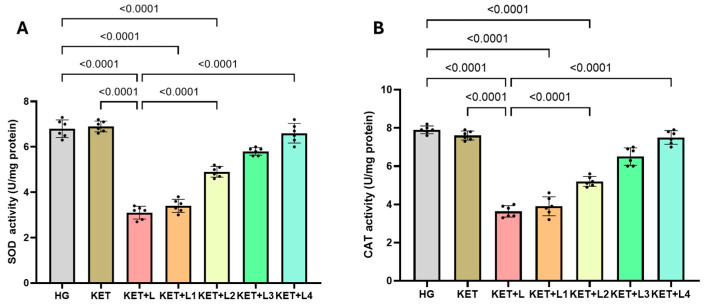
Blood SOD (**A**) and CAT (**B**) activities (U/mg protein) in the experimental groups. Bars indicate mean ± standard deviation (SD); *n* = 6 per group. Black dots represent individual animal values. Statistical comparisons between groups were performed using one-way ANOVA followed by Tukey’s post hoc test. Exact *p*-values are shown above the bars. Statistical significance was set at *p* < 0.05. Abbreviations: SOD, superoxide dismutase; CAT, catalase; HG, healthy group; KET, ketamine group; KET + L, laparotomy + ketamine group; KET + L1–L4, laparotomy + repeated ketamine dose groups.

In tail-vein blood samples, SOD activity (U/mg protein) was significantly lower in the KET + L group (3.10 ± 0.28) than in the HG (6.80 ± 0.39) and KET (6.90 ± 0.24) groups (both *p* < 0.0001). Relative to KET + L, SOD activity increased significantly in KET + L2 (4.90 ± 0.24), KET + L3 (5.80 ± 0.17), and KET + L4 (6.60 ± 0.43) (all *p* < 0.001), while a modest increase was observed in KET + L1 (3.40 ± 0.29) (*p* < 0.05 vs. KET + L) (Figure 2A, Table 1).

Similarly, CAT activity (U/mg protein) in tail-vein blood was significantly lower in KET + L (3.63 ± 0.30) than in HG (7.90 ± 0.20) and KET (7.60 ± 0.24) (*p* < 0.0001). Compared with KET + L, CAT activity showed a stepwise significant increase in KET + L2 (5.20 ± 0.26), KET + L3 (6.50 ± 0.46), and KET + L4 (7.50 ± 0.36) (all *p* < 0.001), whereas KET + L1 (3.90 ± 0.50) exhibited a borderline increase (*p* < 0.05) (Figure 2B, Table 1).

### 2.3. Blood Inflammatory Cytokine (IL-1β, IL-6, and TNF-α) Results

**Figure 3:**
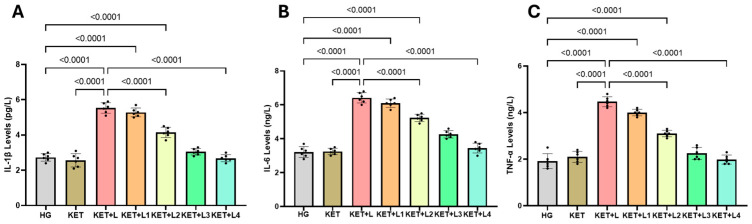
Blood levels of IL-1β (**A**), IL-6 (**B**), and TNF-α (**C**) in the experimental groups. Bars indicate mean ± standard deviation (SD); *n* = 6 per group. Black dots represent individual animal values. Statistical comparisons between groups were performed using one-way ANOVA followed by Tukey’s post hoc test. Exact *p*-values are shown above the bars. Statistical significance was set at *p* < 0.05. Abbreviations: IL-1β, interleukin-1 beta; IL-6, interleukin-6; TNF-α, tumor necrosis factor-alpha; HG, healthy group; KET, ketamine group; KET + L, laparotomy + ketamine group; KET + L1–L4, laparotomy + repeated ketamine dose groups.

In tail-vein blood samples, IL-1β levels (pg/L) were significantly higher in the KET + L group (5.53 ± 0.30) than in the HG (2.73 ± 0.22) and KET (2.57 ± 0.38) groups (both *p* < 0.0001). Relative to KET + L, IL-1β decreased progressively and significantly in KET + L2 (4.15 ± 0.29), KET + L3 (3.05 ± 0.19), and KET + L4 (2.68 ± 0.20) (all *p* < 0.001). No clear reduction was observed in KET + L1 (5.28 ± 0.25) (*p* > 0.05) (Figure 3A, Table 2).

Similarly, blood IL-6 levels (ng/L) were highest in KET + L (6.42 ± 0.31), significantly exceeding HG (3.22 ± 0.32) and KET (3.25 ± 0.19) (*p* < 0.0001). Following repeated ketamine administrations, IL-6 declined progressively in KET + L2 (5.23 ± 0.22), KET + L3 (4.27 ± 0.22), and KET + L4 (3.45 ± 0.29) compared with KET+L (all *p* < 0.001). The difference between KET + L1 (6.10 ± 0.24) and KET + L was minimal and not significant (*p* > 0.05) (Figure 3B, Table 2).

TNF-α levels (ng/L) likewise increased markedly in KET + L (4.47 ± 0.22) relative to HG (1.92 ± 0.32) and KET (2.10 ± 0.24) (*p* < 0.0001). Compared with KET + L, significant reductions were observed in KET + L2 (3.10 ± 0.14), KET + L3 (2.25 ± 0.26), and KET + L4 (1.98 ± 0.19) (all *p* < 0.001), with values in KET + L3 and KET + L4 approaching those of the healthy group (Figure 3C, Table 2).

### 2.4. Blood Catecholamine (ADR and NDR) Results

**Figure 4:**
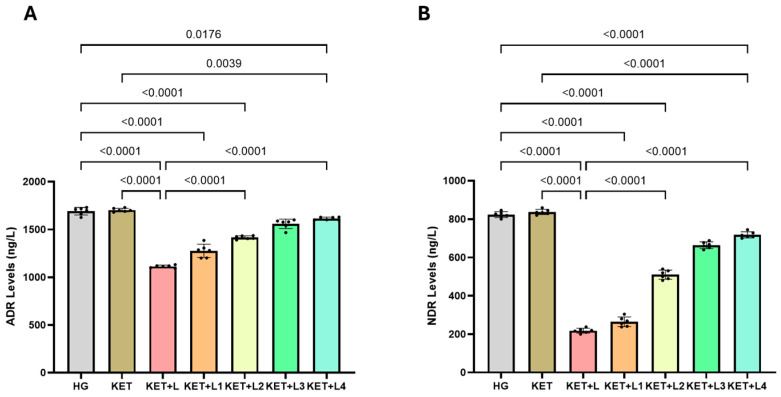
Blood levels of ADR (**A**) and NDR (**B**) in the experimental groups. Bars indicate mean ± standard deviation (SD); *n* = 6 per group. Black dots represent individual animal values. Statistical comparisons between groups were performed using one-way ANOVA followed by Tukey’s post hoc test. Exact *p*-values are shown above the bars. Statistical significance was set at *p* < 0.05. Abbreviations: ADR, adrenaline; NDR, noradrenaline; HG, healthy group; KET, ketamine group; KET + L, laparotomy + ketamine group; KET + L1–L4, laparotomy + repeated ketamine dose groups.

In tail-vein blood samples, adrenaline (ADR; ng/L) levels were significantly lower in the KET + L group (1112.00 ± 13.15) than in the HG (1674.50 ± 38.39) and KET (1703.33 ± 18.31) groups (both *p* < 0.0001). Compared with KET + L, ADR increased progressively and significantly in KET + L1 (1276.67 ± 69.24), KET + L2 (1416.00 ± 16.00), KET + L3 (1559.00 ± 21.91), and KET + L4 (1614.00 ± 14.52) (all *p* < 0.0001) (Figure 4A, Table 3).

Similarly, noradrenaline (NDR; ng/L) levels were markedly lower in KET + L (218.00 ± 13.42) than in HG (823.00 ± 16.97) and KET (837.00 ± 14.65) (*p* < 0.0001). Relative to KET + L, NDR rose significantly in KET + L1 (264.50 ± 25.67), KET + L2 (506.67 ± 20.66), KET + L3 (664.00 ± 17.78), and KET + L4 (718.00 ± 16.91) (all *p* < 0.0001) (Figure 4B, Table 3).

## 3. Discussion

In this study, we investigated the relationship between the duration of intravenous ketamine anesthesia and postoperative trauma–related oxidative stress, inflammatory response, and catecholamine levels in rats. The postoperative trauma model in animals was established via laparotomy. In animal experiments, laparotomy—surgically opening the abdominal wall to access the peritoneal cavity—is commonly used to model surgical trauma [24]. Several sources have reported increases in both oxidative-stress and inflammatory-response markers following laparotomy [25,26,27]. Oxidative stress begins with the intensification of lipid peroxidation (LPO) reactions in the cell membrane [28]. Malondialdehyde (MDA), a highly reactive oxidant molecule produced as a result of LPO, is an important biomarker used to determine long-term cellular damage and oxidative stress [29]. In blood samples collected from the tail vein of the group that underwent laparotomy under single-dose ketamine anesthesia, MDA levels were significantly higher than in the healthy group, indicating the development of oxidative stress in the postoperative period. Our experimental findings are consistent with those of An and colleagues, who demonstrated in a rat brain model that surgical trauma augments LPO reactions [29]. Elevated oxidative-stress parameters in the postoperative period have been linked to complication development and proposed as potential risk-stratification biomarkers [30]. Indeed, both clinical and preclinical studies have shown that surgical trauma triggers oxidative stress [13,29,31,32]. For this reason, postoperative trauma is emphasized as a systemic pathophysiological process that disrupts tissue integrity [7]. Our findings (a decrease in MDA levels; increases in tGSH, SOD and CAT levels; and normalization of these parameters toward control values with prolonged or repeated ketamine administration) suggest that ketamine attenuates oxidative burden in a time-dependent manner. Potential mechanisms include reduced calcium influx due to NMDA-receptor blockade, which in turn limits mitochondrial ROS production, as well as activation of the Nrf2/Keap1 pathway, leading to enhancement of endogenous antioxidant defenses such as the SOD, CAT and GSH systems. In parallel, suppression of the inflammatory response via the NF-κB/MAPK axis is expected to reduce lipid peroxidation and consequently lower MDA levels [19,20]. This mechanistic framework is consistent with the marked improvements observed in the KET + L4 group in our study (e.g., a decrease in MDA from 3.90 ± 0.45 to 2.30 ± 0.26; an increase in SOD from 3.10 ± 0.28 to 6.60 ± 0.43; and an increase in CAT from 3.63 ± 0.30 to 7.50 ± 0.36).

Living organisms have evolved comprehensive endogenous antioxidant defense systems to prevent ROS formation or limit their harmful effects [33]. In states of oxidative stress induced by surgical trauma, depletion of GSH stores and the presence of an impaired antioxidant mechanism are well recognized [31,34]. GSH is a non-protein intracellular thiol and the principal non-enzymatic antioxidant capable of detoxifying hydroxyl radicals (OH–) by electron donation [34]. In our study, total GSH (tGSH) levels in blood samples from animals subjected to laparotomy under single-dose ketamine anesthesia were significantly lower than in the healthy group. Parallel to the reduction in tGSH, activities of the antioxidant enzymes SOD and CAT were also decreased. As is known, SOD and CAT are key antioxidant enzymes located in the mitochondria and cytosol, and they participate in ROS scavenging [35]. SOD converts the superoxide radical to hydrogen peroxide, while CAT decomposes hydrogen peroxide into water and oxygen [35]. Our findings indicate that the oxidative defense system is markedly suppressed following laparotomy. Similarly, Saimanen and colleagues reported plasma SOD and CAT levels were decreased in patients undergoing laparotomy [36]. In another study, reported that ovariohysterectomy via midline laparotomy reduced serum SOD activity in rats [37].

From an inflammatory perspective, we observed significant increases in IL-1β, IL-6 and TNF-α levels in the group that underwent laparotomy under single-dose ketamine anesthesia compared with the healthy group. The rise in pro-inflammatory cytokines after surgical trauma may herald a systemic inflammatory response [38]. In particular, cytokines such as IL-1β, IL-6 and TNF-α are known to be directly associated with postoperative complications and to play central roles in the development of multiple organ dysfunction [7]. Consistent with prior reports, in patients with sepsis or septic shock undergoing emergency laparotomy, peritoneal concentrations of IL-1β, IL-6 and TNF-α were significantly higher than in non-septic patients [38]. Likewise Müsri and colleagues demonstrated that serum TNF-α, IL-1β and IL-6 levels were substantially elevated in rats subjected to an intestinal ischemia/reperfusion model created by laparotomy [39]. Our data align with these findings and support the notion that surgical trauma triggers the inflammatory response.

We also evaluated catecholamine levels, which are biochemical indicators relevant to postoperative hypotension—a recognized complication of surgical trauma. In the group that underwent laparotomy under single-dose ketamine anesthesia, blood adrenaline (ADR) and noradrenaline (NDR) levels were significantly reduced. Physiologically, acute surgical stress is expected to activate the sympathoadrenal system and increase catecholamine release [14], aiming to sustain tissue perfusion by raising heart rate and blood pressure [40,41]. However, in trauma, factors such as hypovolemia, tissue hypoxia, and systemic inflammation may suppress adrenal catecholamine release [42]. Surgery-related decreases in ADR and NDR can predispose to serious hemodynamic complications, including hypotension, bradycardia, and organ hypoperfusion [10,12,43]. Postoperative hypotension is frequent yet easily overlooked [11]. Our findings thus partially contradict the prevailing literature suggesting that surgical trauma enhances sympathoadrenal activity [14,40,43].

Taken together, our results indicate that laparotomy can induce oxidative stress, provoke an inflammatory response, and precipitate hemodynamic disturbances. Reducing postoperative complications is therefore of critical importance [1,6,8,10,12]. Our experimental data, together with existing literature, indicate that the choice of anesthetic agent can substantially influence postoperative trajectories [14]. While some studies emphasize ketamine’s antioxidant properties, others report increases in oxidative-stress parameters. Ghaffari and colleagues showed that ketamine elevated the activities of antioxidant enzymes such as SOD and CAT in rat brain [19]. Similarly, another previous study reported that ketamine reduced MDA levels and increased SOD activity in a brain ischemia model [20]. In contrast, Bedir and colleagues demonstrated that ketamine increased oxidant MDA and caused significant decreases in antioxidant tGSH in rat liver tissue [44]. In our study, single-dose ketamine alone did not produce significant biochemical alterations compared with the healthy group. When laparotomy was added to a single dose of ketamine, MDA levels increased; however, with repeated dosing of ketamine, MDA decreased progressively, whereas tGSH levels and SOD and CAT activities rose in parallel. Notably, in the laparotomy group receiving four ketamine doses (KET + L4), these values approached those of the healthy group. This pattern suggests that ketamine’s antioxidant effect may be dependent on duration of administration. While these findings do not overlap with studies proposing a pro-oxidant effect of ketamine [44], they are consistent with studies highlighting its antioxidant and tissue-protective properties [19,20].

Regarding the inflammatory response, pro-inflammatory cytokine levels increased in animals subjected to laparotomy under single-dose ketamine anesthesia, whereas single-dose ketamine alone did not produce significant changes. The progressive decrease in pro-inflammatory cytokines with repeated ketamine dosing indicates a linkage to anesthesia duration. IL-6, which peaked in the single-dose ketamine plus laparotomy group, decreased markedly in the group that received four post-laparotomy doses at appropriate intervals. Both clinical and preclinical studies have underscored ketamine’s anti-inflammatory effects. A prior study showed that ketamine reduced pro-inflammatory IL-6 levels and mitigated systemic inflammatory response syndrome in adult and pediatric patients undergoing cardiopulmonary bypass [45]. Similarly, Spencer and colleagues demonstrated that intravenous ketamine infusion reduced TNF-α and IL-6 levels in rats [46]. In contrast, Bedir and colleagues reported that ketamine increased IL-6 and TNF-α in rat liver tissue [44]. Our findings conflict with those indicating that ketamine triggers the inflammatory response, while aligning with studies highlighting its anti-inflammatory actions [45,46].

Furthermore, in our study, while laparotomy lowered catecholamine levels, repeated ketamine dosing restored ADR and NDR toward normal, pointing to a potential modulatory effect of this anesthetic on the sympathoadrenal system. Although the oxidative milieu and elevated pro-inflammatory cytokines induced by surgical trauma might be expected to enhance sympathetic activation [7], our findings run counter to this general assumption. In this respect, ketamine’s effects may be crucial for reducing intraoperative and postoperative hemodynamic complications such as hypotension [47]. Aksoy and colleagues showed that ketamine significantly increased endogenous ADR and NDR in rats [48]. In a more recent study, Ahiskalioglu and colleagues similarly reported that ketamine elevated serum adrenaline levels in rats [21]. Our findings are consistent with these reports.

Moreover, although the ketamine–xylazine combination is widely used in rat experiments, xylazine’s α2-adrenergic effects may lead to bradycardia, hypotension, reduced catecholamine release, and potentially altered oxidative/inflammatory profiles [49]. In the present study, we used ketamine alone to allow a clearer evaluation of its duration- and repetition-dependent effects, free from potential confounding influences. Future studies comparing ketamine alone versus the ketamine–xylazine combination in terms of redox balance, inflammation, and hemodynamic parameters would provide valuable insights.

In this context, our findings suggest that the balance between oxidative and antioxidative status under surgical stress is sensitive to ketamine exposure duration at a given dose. Consistent with our dataset, ketamine alone (60 mg/kg, i.p.) did not differ from healthy controls in redox indices, whereas a single dose combined with laparotomy was associated with oxidative/inflammatory activation; by contrast, repeating the same dose at 20 min intervals shifted the profile toward lower lipid peroxidation and higher endogenous antioxidant activity, with concomitant cytokine suppression and catecholamine restoration (e.g., MDA 3.90 → 2.30 nmol/mL; SOD 3.10 → 6.60 U/mg; CAT 3.63 → 7.50 U/mg; IL-6 6.42 → 3.45 ng/L; TNF-α 4.47 → 1.98 ng/L; ADR 1112 → 1614 ng/L; NDR 218 → 718 ng/L). These results align with reports that ketamine’s redox impact may be dose- and time-dependent, potentially reflecting interactions among NMDA-receptor blockade, Nrf2-linked antioxidant responses, and modulation of inflammatory signaling [20,22,23,45].

## 4. Methods and Materials

### 4.1. Animals

A total of 42 male albino Wistar rats weighing 263–277 g were used in the experiment. The animals were obtained from the Erzincan Binali Yıldırım University Experimental Animals Application and Research Center (Erzincan, Türkiye). The animals were randomly allocated to seven groups, ensuring similar mean body weights across groups. Prior to the experiment, to facilitate acclimatization to laboratory conditions, rats were housed in standard wire laboratory cages in groups of six (height: 20 cm, width: 35 cm, length: 55 cm; floor area: 1925 cm^2^). Rats were maintained under controlled environmental conditions with a 12 h light/12 h dark cycle, a constant temperature of 22 °C, and relative humidity of 30–70%. Throughout the study, tap water and commercially available pellet feed (laboratory animal feed; Bayramoğlu Hayvancılık A.Ş., Erzurum, Türkiye) were provided. The study was conducted in accordance with European Directive 2010/63/EU on the protection of animals used for scientific purposes (Approval No: 2016-24-199), and the ARRIVE (Animal Research: Reporting of In Vivo Experiments) guidelines were followed [50]. Experimental procedures were performed in the laboratories of the Erzincan Binali Yıldırım University Experimental Animals Application and Research Center. All procedures involving animals received prior approval from the Erzincan Binali Yıldırım University Local Animal Experiments Ethics Committee (Erzincan, Türkiye; Meeting Date: 29 May 2025; Meeting No: 2025/05; Approval No: 26). The protocols and procedures were also approved by the local Animal Experimentation Ethics Committee (Date: 26 June 2025; Meeting No: 2025/06). Group allocation was known to personnel performing the interventions (e.g., surgical procedures and ketamine dosing), as blinding at this stage was not feasible due to the nature of the protocol. However, to minimize assessment bias, outcome assessors responsible for biochemical and cytokine measurements were blinded to group assignments. Data analysis was performed using coded group labels, and the analyst was not informed of treatment conditions until after statistical evaluation was completed.

### 4.2. Chemicals

Ketamine used in the experiment was obtained from Pfizer Pharmaceuticals Ltd. Şti. (İstanbul, Türkiye).

### 4.3. Experimental Groups

Animals were divided into seven groups, namely healthy control (HG) and those receiving ketamine at a dose of 60 mg/kg (KET, KET + L, KET + L1, KET + L2, KET + L3, and KET + L4).

### 4.4. Experimental Procedure

Rats in the KET, KET + L (*n* = 6), KET + L1 (*n* = 6), KET + L2 (*n* = 6), KET + L3 (*n* = 6), and KET + L4 (*n* = 6) groups received ketamine at a dose of 60 mg/kg [20,48] via intraperitoneal (i.p.) injection. After ketamine administration, the animals were observed until a surgical plane of anesthesia was achieved. The period during which the animals remained immobile in the supine position was considered a surgical plane of anesthesia [51]. During this period, under sterile conditions, rats (except for the KET group) underwent a laparotomy via a 2.5 cm midline incision to access the abdominal cavity. The incision was then closed with sterile surgical sutures. The laparotomy procedure was completed within fifteen minutes. Following laparotomy, ketamine was administered i.p. at the same dose at 20 min intervals as follows: once in the KET + L1 group, twice in the KET + L2 group, three times in the KET + L3 group, and four times in the KET + L4 group. In the HG (*n* = 6) group, distilled water was administered as the vehicle. After administration of ketamine and vehicle, the animals were kept under normal laboratory conditions for 24 h. At the end of this period, all animals were euthanized under high-dose ketamine anesthesia (120 mg/kg) [52]. Immediately prior to euthanasia, blood samples were collected from the tail vein to measure malondialdehyde (MDA), total glutathione (tGSH), superoxide dismutase (SOD), catalase (CAT), IL-1β, IL-6, TNF-α, adrenaline (ADR), and noradrenaline (NDR) levels. Experimental results obtained from all groups were compared with one another.

### 4.5. Biochemical Analysis Methods

#### 4.5.1. Determination of MDA, GSH, SOD and CAT

MDA, total GSH, and SOD in blood were determined according to the manufacturers’ instructions using the following kits: MDA (Product No. 10009055), total GSH (Product No. 703002), and SOD (Product No. 706002), all from Cayman Chemical Co. (Ann Arbor, MI, USA). CAT activity was determined according to the method described by Goth [53]. Protein concentrations were determined using the Bradford assay, which relies on the binding of Coomassie Brilliant Blue G-250 dye to proteins; absorbance of the resulting complex was measured spectrophotometrically at 595 nm [54].

#### 4.5.2. Measurement of ADR and NDR Levels in Rats

Blood samples were collected from the hearts of rats into 2 mL EDTA vacuum tubes to determine adrenaline and noradrenaline levels. Within 15 min of venesection, the EDTA samples for adrenaline and noradrenaline measurements were placed on ice and centrifuged at 3500× *g* for 5 min. After centrifugation, plasma adrenaline and noradrenaline concentrations were measured by an isocratic system using a high-performance liquid chromatography (HPLC) pump (Hewlett Packard Agilent 1100; flow rate: 1 mL/min; injection volume: 40 μL; analytical run time: 20 min) and an electrochemical detector. A reagent kit for HPLC analysis of plasma/serum catecholamines was used (Chromsystems, Munich, Germany).

#### 4.5.3. Blood TNF-α, IL-1β, and IL-6 Determination

Levels of tumor necrosis factor-alpha (TNF-α; ng/L), interleukin-1 beta (IL-1β; pg/L), and interleukin-6 (IL-6; ng/L) were measured using commercial ELISA kits supplied by Eastbiopharm Co. Ltd. (Hangzhou, China), according to the manufacturer’s instructions.

### 4.6. Statistical Analysis—Methods

Data are presented as mean ± standard deviation (*n* = 6 per group). The assumption of normality was assessed using the Shapiro–Wilk test, and homogeneity of variances was evaluated with Levene’s test. For each parameter, one-way ANOVA was applied to compare groups. When significant differences were found, multiple comparisons were performed using Tukey’s Honest Significant Difference (HSD) test. Figure preparation was performed using GraphPad Prism 10.5.0 (GraphPad Software, San Diego, CA, USA, 2025). A two-sided *p* < 0.05 was considered statistically significant.

## 5. Conclusions

In this experimental study, we examined the relationship between the duration-dependent effects of ketamine and surgical-trauma-induced oxidative stress, inflammatory response, and catecholamine (ADR, NDR) levels. The findings show that laparotomy triggers pronounced oxidative and inflammatory activation; the accompanying decreases in ADR and NDR suggest suppression of the sympathoadrenal response.

As ketamine duration/repeat dosing increased, the biological stress response was significantly modulated: MDA decreased; tGSH, SOD and CAT increased; IL-1β, IL-6 and TNF-α declined; and ADR and NDR approached physiological ranges. This pattern suggests that increases in oxidant/inflammatory burden may be associated with decreases in catecholamines, whereas suppression of this burden with ketamine coincides with restoration of catecholaminergic tone.

These results indicate that, beyond its anesthetic actions, ketamine has the potential to regulate postoperative biological stress responses. The decisive roles of administration duration and dosing argue for a duration-sensitive reappraisal of ketamine use in perioperative protocols. Nevertheless, to translate these findings into clinical practice, further preclinical and clinical studies encompassing different surgical models and patient profiles are required. Detailing the duration-dependent effects of ketamine on oxidative stress, inflammation, and hemodynamic balance—and evaluating strategies for targeted modulation of catecholamine levels in the postoperative period—may guide improvements in surgical safety and patient recovery.

In this model, laparotomy performed under a single dose of ketamine induced marked oxidative and inflammatory activation, whereas increasing the duration or repetition of ketamine administration (particularly four doses at 20 min intervals) reduced oxidative burden, enhanced antioxidant defense mechanisms, and restored ADR/NDR levels toward physiological ranges. Therefore, in experimental surgical models, prolonged or repeated ketamine anesthesia appears more suitable than single-dose administration for preserving animal health. However, the generalizability of this recommendation should be validated by further preclinical and clinical studies in different surgical contexts and species.

This study has several limitations. Findings from animal models cannot always be directly extrapolated to human physiology. The molecular signaling pathways underlying ketamine’s effects (e.g., NF-κB, Nrf2, MAPK) were not assessed here; therefore, inferences about the cellular mechanisms underlying the observed biochemical improvements are limited. Another limitation is the lack of molecular-level investigations such as ROS quantification, ROS-induced apoptosis assays, and gene-expression analyses related to oxidative stress and inflammation. Future studies employing these methods will be essential to confirm and extend the present biochemical findings at the cellular and transcriptional levels. Additionally, our anesthesia protocol employed ketamine at 60 mg/kg (i.p.) as a conventional induction dose to achieve surgical depth for midline laparotomy in adult male Wistar rats. While appropriate within species-specific experimental practice, anesthetic requirements are strain-, sex-, age-, and procedure-dependent; therefore, rodent mg/kg values are not directly translatable to human clinical dosing [20,48]. Accordingly, the translational interpretation of our findings emphasizes biological directionality (oxidative/inflammatory load and catecholaminergic tone) and exposure-duration effects rather than absolute dose equivalence across species. Moreover, we did not conduct a formal multi-dose/route titration, which may limit generalizability of the dose rationale to other experimental conditions.

## Figures and Tables

**Table 1:** Effects of intravenous ketamine anesthesia duration on blood oxidative stress and antioxidant parameters in rats subjected to postoperative trauma.

Biochemical Variables				
Groups	MDA (nmol/mL)	tGSH (nmol/mL)	SOD (U/mg Protein)	CAT (U/mg Protein)
HG	2.10 ± 0.36	4.50 ± 0.37	6.80 ± 0.39	7.90 ± 0.20
KET	2.00 ± 0.46	4.70 ± 0.31	6.90 ± 0.24	7.60 ± 0.24
KET + L	3.90 ± 0.45 *^,†^	1.80 ± 0.29 *^,†^	3.10 ± 0.28 *^,†^	3.63 ± 0.30 *^,†^
KET + L1	3.70 ± 0.44 *^,†^	2.20 ± 0.21 *^,†^	3.40 ± 0.29 *^,†,‡^	3.90 ± 0.50 *^,†,‡^
KET + L2	2.80 ± 0.42 *^,‡^	2.90 ± 0.24 *^,†,‡^	4.90 ± 0.24 *^,†,‡^	5.20 ± 0.26 *^,†,‡^
KET + L3	2.50 ± 0.39 ^‡^	3.40 ± 0.29 ^‡^	5.80 ± 0.17 ^‡^	6.50 ± 0.46 ^‡^
KET + L4	2.30 ± 0.26 ^‡^	3.90 ± 0.35 ^‡^	6.60 ± 0.43 ^‡^	7.50 ± 0.36 ^‡^

Footnotes: The results are presented as mean ± standard deviation. For all groups *n* = 6. * *p* < 0.05 vs. HG; ^†^ *p* < 0.05 vs. KET; ^‡^ *p* < 0.05 vs. KET + L. Abbreviations: HG: Healthy group; KET: ketamine group; KET + L: ketamine + laparotomy group; KET + L1–L4: ketamine + laparotomy with repeated ketamine doses; MDA: malondialdehyde; tGSH: total glutathione; SOD: superoxide dismutase; CAT: catalase.

**Table 2:** Effects of the duration of intravenous ketamine anesthesia on blood inflammatory cytokine levels in rats subjected to postoperative trauma.

Biochemical Variables			
Groups	IL-1β (pg/L)	IL-6 (ng/L)	TNF-α (ng/L)
HG	2.73 ± 0.22	3.22 ± 0.32	1.92 ± 0.32
KET	2.57 ± 0.38	3.25 ± 0.19	2.10 ± 0.24
KET + L	5.53 ± 0.30 *^†^	6.42 ± 0.31 *^†^	4.47 ± 0.22 *^†^
KET + L1	5.28 ± 0.25 *	6.10 ± 0.24 *	4.00 ± 0.14 *
KET + L2	4.15 ± 0.29 *^‡^	5.23 ± 0.22 *^‡^	3.10 ± 0.14 *^‡^
KET + L3	3.05 ± 0.19 ^‡^	4.27 ± 0.22 ^‡^	2.25 ± 0.26 ^‡^
KET + L4	2.68 ± 0.20 ^‡^	3.45 ± 0.29 ^‡^	1.98 ± 0.19 ^‡^

Footnotes: The results are presented as mean ± standard deviation. For all groups *n* = 6. * *p* < 0.05 vs. HG; ^†^ *p* < 0.05 vs. KET; ^‡^ *p* < 0.05 vs. KET + L. Abbreviations: HG: Healthy group; KET: ketamine group; KET + L: ketamine + laparotomy group; KET + L1–L4: ketamine + laparotomy with repeated ketamine doses; IL-1β: interleukin-1 beta; IL-6: interleukin-6; TNF-α: tumor necrosis factor-alpha.

**Table 3:** Effects of intravenous ketamine anesthesia duration on blood catecholamine levels in rats subjected to postoperative trauma.

Biochemical Variables		
Groups	ADR (ng/L)	NDR (ng/L)
HG	1674.50 ± 38.39	823.00 ± 16.97
KET	1703.33 ± 18.31	837.00 ± 14.65
KET + L	1112.00 ± 13.15 *^†^	218.00 ± 13.42 *^†^
KET + L1	1276.67 ± 69.24 *^‡^	264.50 ± 25.67 *^‡^
KET + L2	1416.00 ± 16.00 *^‡^	506.67 ± 20.66 *^‡^
KET + L3	1559.00 ± 21.91 ^‡^	664.00 ± 17.78 ^‡^
KET + L4	1614.00 ± 14.52 ^‡^	718.00 ± 16.91 ^‡^

Footnotes: The results are presented as mean ± standard deviation. For all groups *n* = 6. * *p* < 0.05 vs. HG; ^†^ *p* < 0.05 vs. KET; ^‡^ *p* < 0.05 vs. KET + L. Abbreviations: HG: Healthy group; KET: ketamine group; KET + L: ketamine + laparotomy group; KET + L1–L4: ketamine + laparotomy with repeated ketamine doses; ADR: adrenaline; NDR: noradrenaline.

## Data Availability

Data from the research can be obtained from the corresponding author upon reasonable request.

## Abbreviations

The following abbreviations are used in this manuscript:

MDA	Malondialdehyde
ADR	Adrenaline
NDR	Noradrenaline
tGSH	Total Glutathione
SOD	Superoxide Dismutase
CAT	Catalase
ROS	Reactive Oxygen Species
LPO	Lipid Peroxidation
MPA	Mean Arterial Pressure
NMDA	N-methyl-D-aspartate
IL-1β	Interleukin-1 Beta
IL-6	Interleukin-6
TNF-α	Tumor Necrosis Factor-Alpha
